# Improving RNA-Seq expression estimates by correcting for fragment bias

**DOI:** 10.1186/gb-2011-12-3-r22

**Published:** 2011-03-16

**Authors:** Adam Roberts, Cole Trapnell, Julie Donaghey, John L Rinn, Lior Pachter

**Affiliations:** 1 Department of Computer Science, 387 Soda Hall, UC Berkeley, Berkeley, CA 94720, USA; 2 Broad Institute, 7 Cambridge Center, Cambridge, MA 02142, USA; 3 Department of Stem Cell and Regenerative Biology, 7 Divinity Avenue, Harvard University, Cambridge, MA 02138, USA; 4 Departments of Mathematics and Molecular & Cell Biology, 970 Evans Hall, UC Berkeley, Berkeley, CA 94720, USA

## Abstract

The biochemistry of RNA-Seq library preparation results in cDNA fragments that are not uniformly distributed within the transcripts they represent. This non-uniformity must be accounted for when estimating expression levels, and we show how to perform the needed corrections using a likelihood based approach. We find improvements in expression estimates as measured by correlation with independently performed qRT-PCR and show that correction of bias leads to improved replicability of results across libraries and sequencing technologies.

## Background

**Figure 1:**
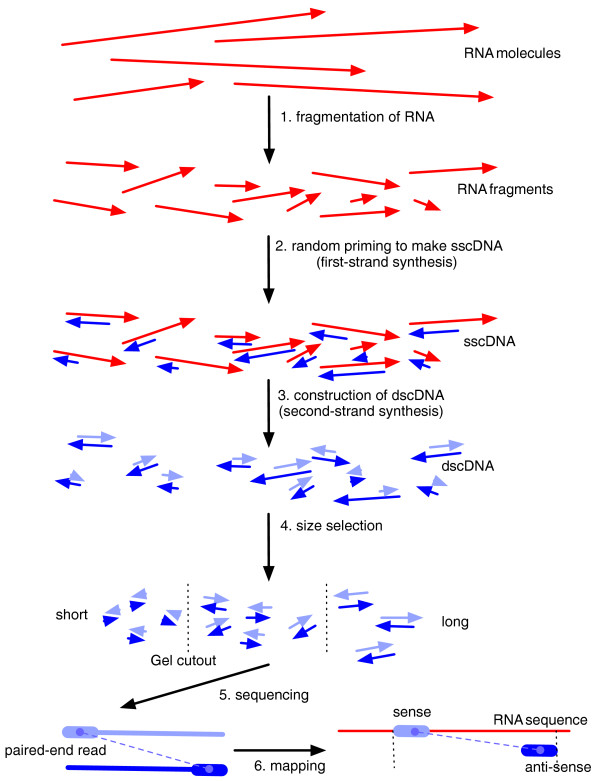
**Overview of a typical RNA-Seq experiment**. RNA is initially fragmented (1) followed by first-strand synthesis priming (2), which selects the 3' fragment end (in transcript orientation), to make single stranded cDNA. Double stranded cDNA created during second-strand synthesis (3), which selects the 5' fragment end, is then size selected (4) resulting in fragments suitable for sequencing (5). Sequenced reads are mapped to opposite strands of the genome (6), and in the case of known transcript or fragment strandedness, the read alignments reveal the 5' and 3' ends of the sequenced fragment (see Supplementary methods in Additional file 3). All arrows are directed 5' to 3' in transcript orientation.

RNA-Seq technology offers the possibility of accurately measuring transcript abundances in a sample of RNA by sequencing of double stranded cDNA [1]. Unfortunately, current technological limitations of sequencers require that the cDNA molecules represent only partial fragments of the RNA being probed. The cDNA fragments are obtained by a series of steps, often including reverse transcription primed by random hexamers (RH), or by oligo (dT). Most protocols also include a fragmentation step, typically RNA hydrolysis or nebulization, or alternatively cDNA fragmentation by DNase I treatment or sonication. Many sequencing technologies also require constrained cDNA lengths, so a final gel cutting step for size selection may be included. Figure 1 shows how some of these procedures are combined in a typical experiment.

The randomness inherent in many of the preparation steps for RNA-Seq leads to fragments whose starting points (relative to the transcripts from which they were sequenced) appear to be chosen approximately uniformly at random. This observation has been the basis of assumptions underlying a number of RNA-Seq analysis approaches that, in computer science terms, invert the 'reduction' of transcriptome estimation to DNA sequencing [2-6]. However, recent careful analysis has revealed both positional [7] and sequence-specific [8,9] biases in sequenced fragments. Positional bias refers to a local effect in which fragments are preferentially located towards either the beginning or end of transcripts. Sequence-specific bias is a global effect where the sequence surrounding the beginning or end of potential fragments affects their likelihood of being selected for sequencing. These biases can affect expression estimates [10], and it is therefore important to correct for them during RNA-Seq analysis.

**Figure 2:**
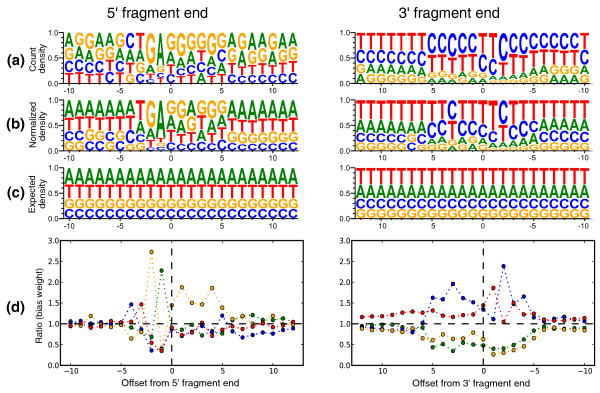
**Nucleotide distribution surrounding fragment ends and calculation of bias weights**. **(a) **Sequence logos showing the distribution of nucleotides in a 23 bp window surrounding the ends of fragments from an experiment primed with 'not not so random' (NNSR) hexamers [11]. The 3' end sequences are complemented (but not reversed) to show the sequence of the primer during first-strand synthesis (see Figure 1). The offset is calculated so that zero is the 'first' base of the end sequence and only non-negative values are internal to the fragment. Counts were taken only from transcripts mapping to single-isoform genes. **(b) **Sequence logo showing normalized nucleotide frequencies after reweighting by initial (not bias corrected) FPKM in order to account for differences in abundance. **(c) **The background distribution for the yeast transcriptome, assuming uniform expression of all single-isoform genes. The difference in 5' and 3' distributions are due to the ends being primed from opposite strands. Comparing (c) to (a) and (b) shows that while the bias is confounded with expression in (a), the abundance normalization reveals the true bias to extend from 5 bp upstream to 5 bp downstream of the fragment end. Taking the ratio of the normalized nucleotide frequencies (b) to the background (c) for the NNSR dataset gives bias weights **(d)**, which further reveal that the bias is partially due to selection for upstream sequences similar to the strand tags, namely TCCGATCTCT in first-strand synthesis (which selects the 5' end) and TCCGATCTGA in second-strand synthesis (which selects the 3' end). Although the weights here are based on independent frequencies, we found correlations among sites in the window and take these into account in our full model to produce more informative weights (see Supplementary methods in Additional file 3). A similar figure to this for the standard Illumina Random Hexamer protocol and plots similar to (d) for all datasets in the paper can be found in Figures S1 and S2 of Additional file 1 respectively.

Although many biases can be traced back to specifics of the preparation protocols (see Figure 2 and [8]), it is currently not possible to predict fragment distributions directly from a protocol. This is due to many factors, including uncertainty in the biochemistry of many steps and the unknown shape and effect of RNA secondary structure on certain procedures [10]. It is therefore desirable to estimate the extent and nature of bias indirectly by inferring it from the data (fragment alignments) in an experiment. However, such inference is non-trivial due to the fact that fragment abundances are proportional to transcript abundances, so that the expression levels of transcripts from which fragments originate must be taken into account when estimating bias, as Figure 2 demonstrates. At the same time, expression estimates made without correcting for bias may lead to the over- or under-representation of fragments. Therefore the problems of bias estimation and expression estimation are fundamentally linked, and must be solved together. Likelihood based approaches are well suited to resolving this difficulty, as the bias and abundance parameters can be estimated jointly by maximizing a likelihood function for the data.

Our main result is the development of a likelihood based approach for simultaneous estimation of bias parameters and expression levels using the likelihood framework of [6]. This complements work of [8,10] where corrections are developed based on another likelihood model, and also extends their work by incorporating simultaneous estimation and correction of positional bias. We demonstrate that our method improves expression estimates in comparison with independently obtained qRT-PCR on a benchmark dataset. Using the same data, we also show that our method improves on the approaches of [8,10]. RNA-Seq technology is changing rapidly, and this is evident in the development of numerous preparation protocols (for a recent review see [11]) and increasingly longer read lengths from sequencing machines [12]. When assessing the impact of bias correction, we have therefore included both early RNA-Seq data of the type that many laboratories might be producing with older machines, as well as newer data that reflects recent protocol choices and demonstrates the improvements in sequencing technologies. This has required us to make our methods robust to both single- and paired-end reads, strand specific and non-specific protocols, and a variety of priming and fragmentation methods. One of our main findings is that bias correction improves the correlation of expression estimates obtained from sequence data generated using different sample preparations and different sequencing technologies.

## Results and Discussion

### Estimating fragment bias in existing protocols

Fragment counts in an RNA-Seq experiment are determined by two different phenomena: fragments originating from highly expressed transcripts will appear more often in the data than those originating from lower-expressed transcripts, and library preparations include biases that may preferentially select some potential fragments over others. By fragment bias we mean only the over- or under-representation of fragments due to sequence-specific or positional bias as discussed in the Background. Because expression levels also affect fragment abundances, it is necessary to jointly estimate transcript abundances and bias parameters in order to properly learn the bias directly from RNA-Seq data.

**Figure 3:**
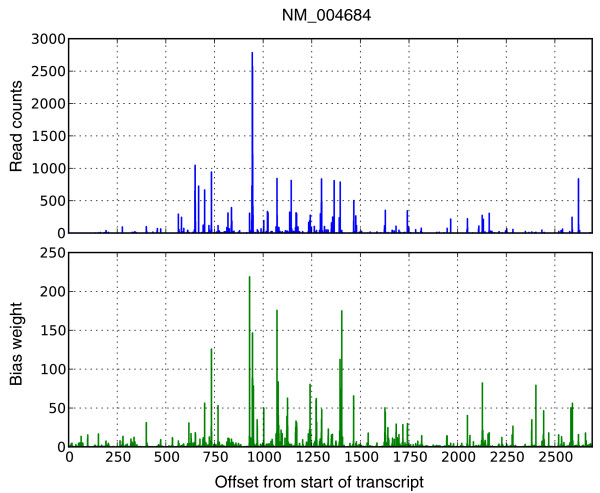
**Bias correction within transcripts**. An example showing the effect of bias correction on the read counts for human transcript NM_004684. The top panel shows raw read counts (number of 3' ends of fragments at each location), and the bottom panel shows the product of the bias parameters (total bias weight defined in the Supplementary methods in Additional file 3) at the same locations. We correctly identify bias at different positions and can therefore correct for the non-uniformity. Note that the bias parameters were learned from the entire dataset excluding reads mapped to this transcript in order to cross-validate our results. The RNA-Seq for the experiment was performed with the NSR protocol [21], which is why 3' counts were used instead of 5'.

This issue is illustrated by example in Figure 2 where the need for joint estimation of bias parameters and expression values is evidenced by comparison of the raw counts of bases at the starts/ends of fragments (panel a) and the adjusted counts normalized by the abundances of transcripts (panel b). The latter calculation is affected by the bias parameters, so that joint estimation is required. We expanded the likelihood framework described in [6] in order to perform such parameter estimation (see Materials and methods), resulting in 'learned' bias weights (panel d Figure 2) that were used to adjust expected fragment counts in the computation of abundances using our likelihood model. Figure 3 shows an example of how well these bias estimates capture the over- and under-representations of reads at different positions of a transcript, based on its sequence.

### Validation by comparison to alternative expression assays

We emphasize that our goal was not to validate RNA-Seq *per se*, but rather to show that bias correction improves expression estimation. Therefore, in interpreting the correlations throughout the paper, we focused on improvements in correlation with bias correction and not on the absolute value. In this regard, we report most of our results as fraction discrepancy explained, which we calculated by dividing the change in *R*^2 ^after bias correction by the difference of the initial *R*^2 ^from 1 (a perfect correlation). Selected correlation plots can be found in Figure S3 of Additional file 1 and all raw expression data in Additional file 2. Furthermore, we mention that we observed that correlation results were sensitive to the extent of filtering of low abundance fragments and we therefore attempted to eliminate filtering in the experiments we performed (see Materials and methods for more detail).

A major problem with validating RNA-Seq expression estimates is that there is no clear 'gold standard' for expression estimation. Comparison of RNA-Seq to microarrays has suggested that the former technology is more accurate than the latter [13]. We examined the recently published NanoString nCounter gene expression system [14], but noticed many unexplainable outliers and high variance between technical replicates (see Figure S4 of Additional file 1 and data in Additional file 2). Quantitative reverse transcription PCR (qRT-PCR) has served as a benchmark in numerous studies but it is not a perfect expression measurement assay [15], and it is therefore *a priori *unclear which technology currently produces the most accurate expression estimates. Nevertheless, at present we believe it to be the best measure of expression aside from, perhaps, RNA-Seq itself. Due to the previously demonstrated superiority of RNA-Seq over microarrays, and the problems with NanoString, we performed all our benchmarking with respect to qRT-PCR.

**Figure 4:**
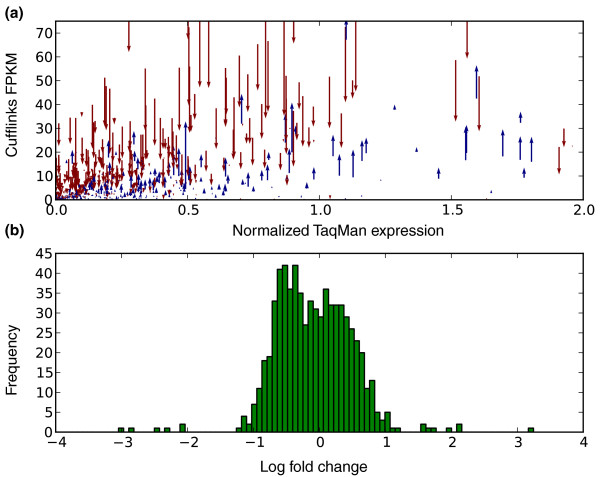
**Correlation between RNA-Seq and qRT-PCR**. **(a) **Expression estimates before bias correction (tail of arrows) and after correction (points of arrows) for the SRA012427 dataset compared to qRT-PCR values for the same transcripts. Red arrows show decrease in expression after correction and blue an increase. Note that we have zoomed in on lower-expression transcripts (the majority) for clarity. **(b) **Distribution of log-fold change in expression after bias correction.

We began by comparing the expression estimates on the Microarray Quality Control (MAQC) Human Brain Reference (HBR) dataset, which includes 907 transcripts with uniquely mapping TaqMan qRT-PCR probes [16], with RNA-Seq data from the same sample sequenced by Illumina (SRA012427) [17] (Figure 4). We examined the correlation of the Cufflinks output with the qRT-PCR expression data and observed an increase of *R*^2 ^from 0.753 before correction to 0.807 after correction.

We examined the basis for change in correlation by further investigating, for each transcript, whether its expression estimate increased or decreased after bias correction, and by how much. The arrows in Figure 4 show the direction and extent of expression change with correction, and the overall fold-change distribution. Many fragments show large changes in expression with a median absolute fold change of 1.5 (Figure 4b). To establish the significance of the improvement in correlation, we performed a permutation test where we changed the expression estimates of transcripts randomly according to the fold change distribution in Figure 4b. We obtained a *P*-value of 0.0007, meaning that the improvement in *R*^2 ^our correction accomplishes is highly significant. Together, these results show that bias correction may dramatically affect expression estimates via both increases and decreases of expression values, and that these changes provide an overall improvement in abundance estimates.

### Comparison with previous methods

**Figure 5:**
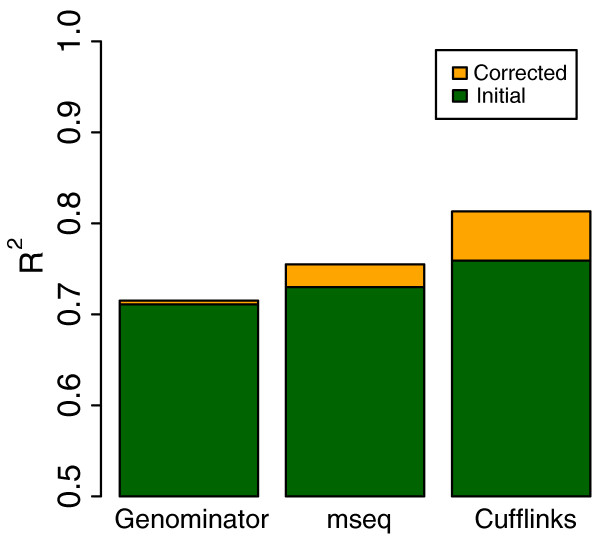
**Comparison with previous methods**. A comparison of our method (Cufflinks) with Genominator[8] and mseq[10]. The *y*-axis shows the *R*^2 ^value for the correlation between uncorrected (green) and bias corrected (orange) RNA-Seq expression estimates and qRT-PCR for the three methods. Correlation plots for these data can be found in Figure S3 of Additional file 1.

In [8], a method for bias correction is proposed that is based on correcting read counts for transcripts according to the bias learned for patterns at the start of reads (normalized using sequences in the interior of reads). This approach uses less information than our method, as it is restricted to learning bias within the read sequence, and cannot capture bias surrounding the start site. Furthermore, count-based methods do not fully exploit the information available in paired-end reads which allow for the determination of fragment length. Fragment length can help in assigning ambiguously mapped fragments to transcripts and our method takes advantage of this. On the other hand, since read counts have been promoted as an acceptable way to measure abundance [18], we compared the method to ours using the MAQC qRT-PCR data from the previous section. Figure 5 shows the results of the method of [8], both before and after bias correction (*R*^2 ^= 0.711 before and *R*^2 ^= 0.715 after correction). To obtain these results we used the software package Genominator[8], following the guidelines in the documentation, with the exception that bias was learned separately for each chromosome, as the software was not able to load an entire genome into memory. More details are provided in the Materials and methods section.

We also compared our approach to the mseq method in [10]. We again used the MAQC HBR qRT-PCR data and this time prepared the sequences and learned parameters for models following the suggested guidelines in [10], that is we trained the parameters of a MART model for bias by learning from the 100 most expressed transcripts in the experiment, and then tested on the set of 907 transcripts with uniquely mapping TaqMan probes. In this case, we observed an uncorrected *R*^2 ^= 0.730 and corrected *R*^2 ^= 0.755. Note that the even though the expression was again calculated using counts, the initial correlation of mseq is better than that of Genominator due to the fact that the implementation in [10] required us to remap the reads directly to the transcript sequences, which is presumably more accurate than relying on spliced mapping.

We suspect that the overall inferior results of both the Genominator and mseq in comparison to Cufflinks are due in part to the fact that the bias parameters cannot be learned from raw read counts, but must be normalized by the expression values of the transcripts from which the reads originate (Figure 2). For example, in [10], bias parameters are learned from what are estimated to be the most highly expressed transcripts based on RPKM, but these are likely to also be the most positively biased transcripts, and are therefore not representative in terms of their sequence content. We also believe that, as we argued in [6], it is important to account for fragment lengths in estimating expression, and read count based expression measures do not use such information. Another issue affecting Genominator is that instead of computing the expected read count as is done in Cufflinks and mseq, the observed read counts are adjusted. This means that in positions lacking read alignments, there is no correction of bias. We believe this may partially explain the improved performance of mseq in comparison to Genominator.

### Technical replicates

A recurring worry with RNA-Seq has been that repeated experiments, possibly based on different libraries or performed in different laboratories, may be variable due to experimental 'noise'. We investigated these effects starting with an exploration of the correlation between technical replicates before and after bias correction. We define technical replicates to be the sequencing of two different libraries that have been prepared using the same protocol from a single sample. This differs slightly from some previous uses; in particular, technical replication has also referred to two sequencing experiments from the same library. Such replicates have already been shown to exhibit very little variability [18,19].

**Figure 6:**
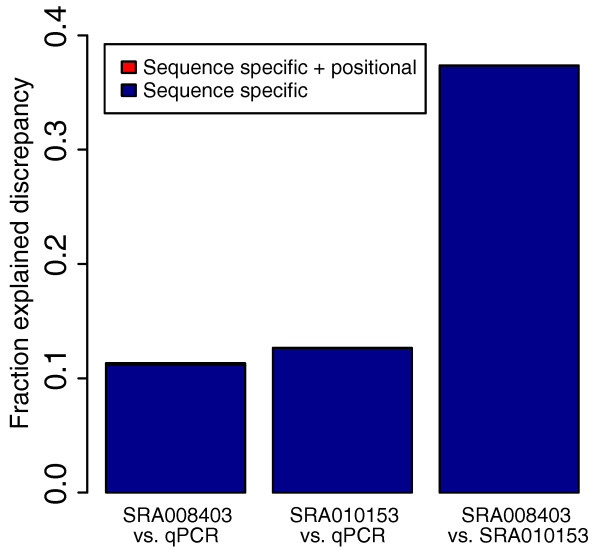
**Variable technical replicates**. Results of correlation tests showing improvement after bias correction for technical replicates. Fraction Explained Discrepancy was calculated by dividing the change in *R*^2 ^after bias correction by the difference of the initial *R*^2 ^from one (a perfect correlation). Note that when two RNA-Seq datasets are compared, the correction in the legend was applied to both. The pairwise correlations of the four SRA010153 replicates versus qRT-PCR and SRA008403, respectively, were averaged for the figure. Even though the same RH priming protocol was used in both labs, the bias differs slightly (see Figure S2 of Additional file 1) between the preps, which is why our correction method was able to improve the correlation.

We postulated that the differences between expression estimates from two different libraries should be reduced after bias correction. We tested this hypothesis in a series of analyses whose results are shown in Figure 6. First, we examined libraries prepared in two different experiments from the same MAQC Universal Human Reference (UHR) sample. In the first experiment [20], which we will refer to by its accession SRA008403, the sample was sequenced from one library preparation. In the second experiment [19], which we will refer to as SRA010153, the sample was sequenced in four separate library preparations. Although the same protocol was used in all five replicates, the learned bias weights differ somewhat between the data produced by the two labs (see Figure S2 in Additional file 1).

Figure 6 shows how correlations of the replicates with qRT-PCR and each other were affected by bias correction. Although the method does improve the pairwise correlations between different library preparations within SRA010153, the initial correlation is already so high (average *R*^2 ^> 0.96) that we only show the average pairwise correlations against qRT-PCR and the SRA008403 dataset. The greater correlation among the SRA010153 replicates as compared to the correlation between them and SRA008403 further indicates that bias is more similar when the protocol is carried out by the same lab, presumably by the same person. Bias correction clearly recovers much of the differences in quantification between the replicates introduced by sequence and positional bias. Furthermore, as in the initial validation example, the correction brings both sets closer in line with the qRT-PCR standard.

### Library preparation methods

**Figure 7:**
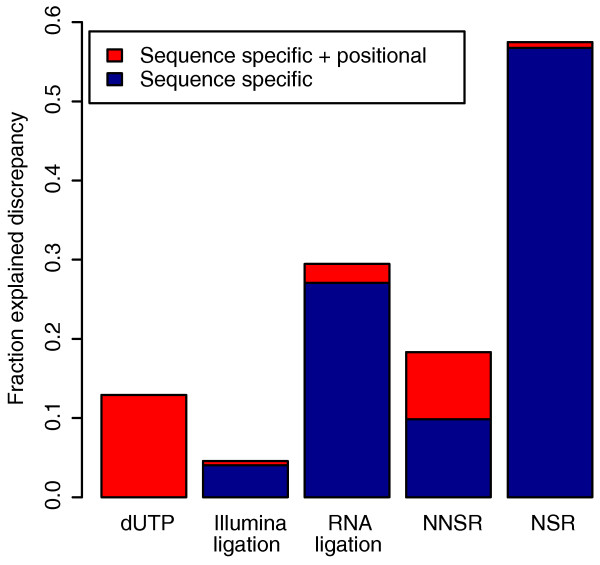
**Variable library preparations**. Results of correlation tests showing improvement after bias correction of datasets generated using different library prep methods, all of which are strand-specific. The first four protocols are described in [11] and the final in [21]. All datasets were compared against a control that was generated using the standard Illumina RH protocol. The first four datasets used the control from [11] with the same yeast sample. The last dataset (NSR) was compared against the HBR dataset from SRA010153 since it is also consists of single-end reads.

In Figure 7 we demonstrate our ability to correct bias specific to libraries prepared using different protocols. For this experiment, we tried our method on several libraries from a study comparing strand-specific protocols (SRA020818) using the same yeast sample [11], as well as a dataset generated using the 'not so random' (NSR) priming protocol on the human MAQC HBR sample [21]. We compared all of these datasets with a standard Random Hexamer (RH) control for the given sample. Note that although the control (RH) and dUTP libraries have the same sequence bias (see Figure S2 in Additional file 1) and near-perfect initial correlation (*R*^2 ^> 0.99), the remaining discrepancy is reduced by positional bias correction.

Because the NSR dataset was sequenced from the MAQC HBR sample, we were also able to compare it to the qRT-PCR standard. We found that our method explained 33.5% of the discrepancy between an initial estimation and qRT-PCR.

### Sequencing platforms

Previous studies on bias in RNA-Seq have focused on experiments performed with Illumina sequencers. To investigate whether bias persists with other prep and sequencing technologies, we examined bias in a SOLiD experiment that sequenced both MAQC samples using the standard whole transcriptome (WT) protocol. We saw clear signs of both sequence-specific and positional bias that differed from the other protocols we had examined (see Figure S2 of Additional le 1).

**Figure 8:**
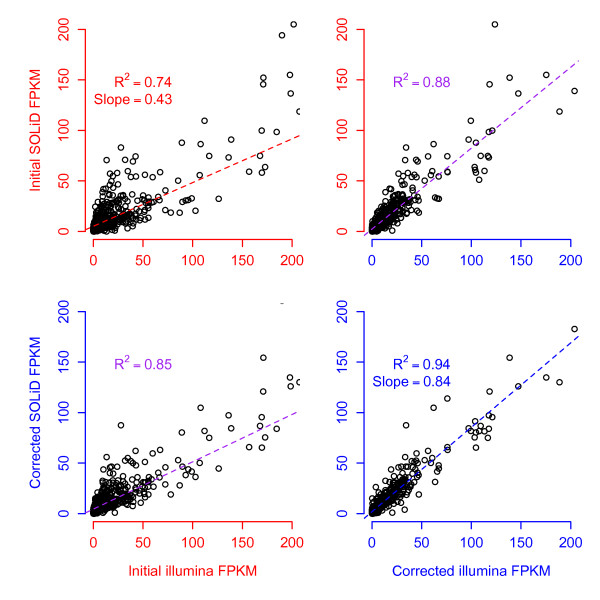
**Bias in different sequence technologies**. Results of correlation tests showing improvement after bias correction of datasets generated using different sequencing technologies. The Illumina dataset is SRA012427 (*x*-axes) and the SOLiD data is SOLiD4_ HBR_PE_50x25 (*y*-axes). Both used the same MAQC HBR sample. Red axes and lines denote uncorrected FPKM values and blue corrected, while purple regression lines denote a comparison between corrected and uncorrected values. Both datasets are being corrected for different biases, which causes their expression estimates to become more correlated. Note that the plot is zoomed in on the lower abundance transcripts for clarity but captures over 98% of those in the experiment.

We next compared the expression estimates for the SOLiD dataset with one from Illumina (accession SRA012427) before and after bias correction. In order to illustrate that our improvement in correlation does not come solely from correcting bias in the Illumina dataset, we tested whether there was some improvement from correcting one dataset at a time, as compared to simultaneous correction for both platforms. We found an increase of *R*^2 ^from 0.74 to 0.88 (Illumina correction) and 0.85 (SOLiD correction) compared to 0.94 for both. These results are summarized in Figure 8. While one cannot draw general conclusions based on a single experiment, we note that our approach to quantifying bias should be useful in future studies that aim to quantitatively compare the bias among different sequencing platforms.

## Conclusions

### Bias correction improves expression estimates

Our results confirm that bias correction improves expression estimates and should be used to correct bias introduced in library preparations and by sequencing technologies. We note that there is great variability in the extent of bias among protocols, and bias correction can dramatically affect expression estimates even in protocols of choice (for example the dUTP protocol currently favored by the Broad Institute [11]).

### Implications for differential expression

It is particularly important to consider bias correction in the context of differential expression analysis. This can refer to the comparison of expression levels among transcripts in a single experiment (for example alternative isoforms of a gene), to the agglomeration of data produced by different laboratories, or to the comparison of expression among biological replicates.

We have shown that bias varies between library preps, even when the same protocol is used. However, our results indicate that this variance is much greater when either different protocols or technologies are used. Therefore, while bias correction can be expected to show small improvements in the former case, it is crucial in modern experiments that seek to combine and compare output from multiple library preps using the same or different protocols. For example, in the Drosophila modENCODE transcriptome experiment described in [22], both SOLiD and Illumina libraries were used at multiple time points during development. To estimate the improvement that could have been gained in the modENCODE experiment by using our correction, we ran Cuffdiff (the differential expression analysis tool packaged with Cufflinks) on the same samples used above to compare bias in the Illumina and SOLiD technologies. We found a 46% decrease in the number of differentially expressed transcripts output by Cuffdiff when bias correction was enabled.

### Choice of model

We have developed a bias correction procedure based on a fragment model for RNA-Seq [6], in contrast with the site model of [10]. We note that our choice is based partly on the observation in [10] that even after bias correction, variability in the counts of reads at individual sites differ considerably from the variance estimate obtained from the binomial model. Thus, it may be that the model of [10] is not robust in multiple isoform genes where few sites may distinguish isoforms. It is likely, however, that as RNA-Seq protocols improve and are better understood, site models will be preferable due to their improved resolution.

**Figure 9:**
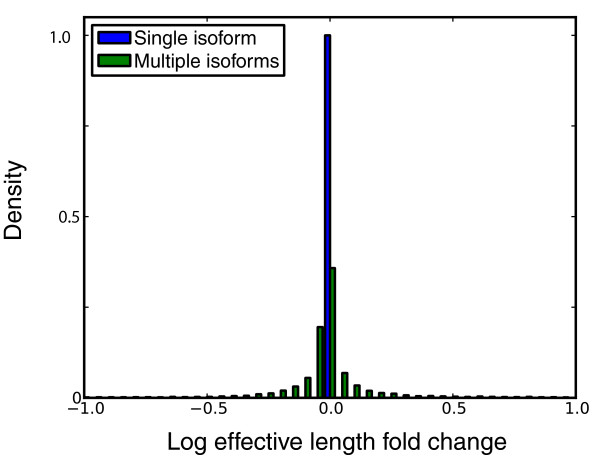
**Positional bias correction effect on expression**. This figure shows the effective length fold change due to positional bias correction for the SRA012427 dataset. So that the parameters would be consistent for all transcripts, we have limited the analysis to transcripts with length greater than 2,433, which is the largest of the 5 length bins we use for measuring positional bias. As expected, all single isoform genes are adjusted in the same way, whereas isoform deconvolution is altered based on the difference in relative position within a transcript for a read that maps to multiple isoforms.

The choice of model has an important implication for the impact of positional bias correction: In our fragment model, positional bias correction without sequence-specific correction does not affect relative expression estimates. However, positional bias correction in multiple isoform genes, or when coupled with sequence-specific correction, can affect relative expression estimates (Figure 9). Validation of the improvement in expression estimates in multiple isoform genes when taking positional bias into account is complicated by the difficulty in selecting isoform specific primers, and is beyond the scope of this paper. It is important to note that in the site model, positional bias correction can affect relative expression estimates even in single isoform genes because the location of fragments within transcripts directly affects the likelihood function.

### GC content and bias

Previous RNA-Seq investigations have revealed correlations between expression levels and GC content, and corrections have been proposed to 'normalize' the data with respect to this effect [23]. When examining the sequence-specific bias profiles (see Figure S2 of Additional file 1) we noticed GC effects in the estimated parameters and so we investigated the relationship between sequence-specific bias correction and GC content.

To make the comparison, we defined the bias of a transcript to be the log fold change in effective length, which is a direct measure of the extent of correction of expression estimate in single isoform genes when incorporating bias correction.

**Figure 10:**
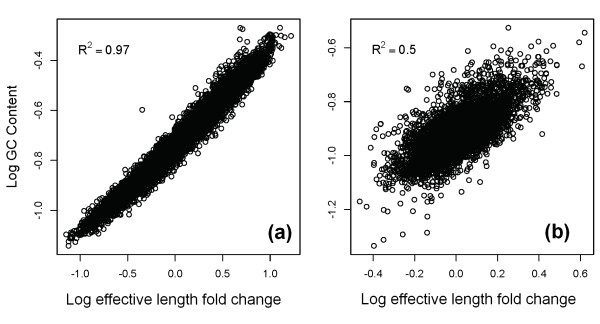
**Correlation of GC content with measured bias**. Panel a shows an example from human and panel b from yeast. Since the log fold change in effective length does not capture the full bias measurement for multiple isoform genes, the plots are limited to those with single isoforms. GC content appears to be correlated with our sequence bias measurements in some datasets, but not in others. GC content alone is not always a good proxy for fragment end bias.

Figure 10 shows the relationship between transcript bias and GC content in two different experiments. In panel a, an example from human RNA-Seq (SRA012427) the correlation is very strong, and shows that GC corrections may be proxies for sequence-specific bias correction. On the other hand, GC content may not always be an effective marker for bias, as shown in panel b from yeast (SRA020818_RH).

We concluded that although normalization of expression values by GC content may be a simple way to remove some bias, it may well be a proxy for other effects rather than of inherent significance.

### Implementation

RNA-Seq data processing pipelines require multiple steps that include read mapping, transcript assembly, expression estimation and differential expression analysis. A difficulty with analysis is that many of these steps are closely related, and improvements in one area can be leveraged in another only if properly integrated. We have shown that in the case of bias correction, estimation of parameters together with abundances can improve expression estimates, and these can in turn affect differential expression analyses, mapping probabilities, and even assemblies.

In order to maximize the benefits of bias correction throughout the RNA-Seq analysis pipeline, we have incorporated it into the Cufflinks RNA-Seq analysis suite [6], and have pre-configured the software for specific protocols so that users can reap the benefits of bias correction for both stranded and unstranded protocols, as well as single- and paired-end reads. The software is freely available [24] and is distributed open source under the Boost Software License, version 1.0.

## Materials and methods

### Parameter estimation and inference

Due to the added sensitivity in our model to the location of fragment ends, we now rely on an empirical fragment length distribution whenever possible, as opposed to the Gaussian approximation in [6]. The fragment length distribution is estimated in one of several ways, depending on what information is provided. If an annotation and paired-end read mappings are given, fragment mappings to single isoform genes are used to determine an empirical distribution. If no annotation is provided, but paired-end read mappings are provided, sufficiently large (≥ 1,000 bp) ranges are found where no fragments have spliced mappings. The mappings in these ranges are used to determine an empirical distribution. If no paired-end fragments are available or not enough are found in these ranges, we use a truncated Gaussian where all lengths less than the minimum read length in the dataset are set to zero probability and the remaining distribution is renormalized. The mean and standard deviation are set according to the distribution specified by the SRA entry, or to 200 and 80, respectively, if the information is unavailable.

The likelihood in our model is a function of the relative transcript abundances (*ρ*), consisting of the abundances for individual transcripts *ρ_t _*such that  (here *T *denotes the set of all transcripts). In order to simplify computations, we estimate the relative abundances for overlapping sets of transcripts instead of directly estimating the parameters *ρ_t_*. We define a locus to be a genomic region containing a set of overlapping transcripts (typically isoforms of a gene) and then write the transcript abundance as *ρ*_*t *_= *β_g_*γ_t _where *β_g _*is the relative abundance of the locus *g *in which *t *is contained, and is multiplied by a factor γ*_t _*that determines the proportion of each transcript within the locus. We denote the set of all loci by *G *(for more details see the Supplementary methods of [6]). Our updated likelihood model, whose full derivation is given in the Supplementary methods in Additional file 3 is then given by:

where *F *is the set of fragments, *X_g _*is the number of fragments with alignments to locus *g*, *I_t_*(*f*) is the implied length of a fragment *f *assuming it originated from a transcript *t *(this is needed because only the ends of fragments are sequenced), *D*(*t *, *f*) is the probability of observing a fragment of length *I_t_*(*f*) at a known position in a transcript, and the term  is the probability of selecting a fragment of a specific length within a given transcript, based on the bias weights at its 5' and 3' end points.

The bias weight *b*(*t*, *i*, *j*) factors as  where *i *and *j *are the 5' and 3' endpoints, respectively, of a fragment mapped to transcript *t*. The  and  weights measure sequence-specific bias and are found by calculating the ratio of the probability of the sequence surrounding the fragment end under the biased model to the uniform (null) model. Note that we model both ends separately due to the differences in sequence selectivity between the priming steps during first- and second-strand synthesis. In our method, these probabilities are actually learned from the data using a variable length Markov model [25] to capture dependencies between positions in the sequence. Complete details are in the Supplementary methods in Additional file 3.

The  and  weights measure the 5' and 3' positional biases, respectively. In [7] it was shown that positional effects depend on transcript length, so we modeled positional effects using 100 = 20 × 5 parameters, with 5 sets of parameters for different transcript lengths (see Figure S2 of Additional file 1). For each range of transcript lengths, the length is divided into 20 windows, each with its own parameter that reflects the probability that the 5' or 3' end of a fragment lies there as opposed to elsewhere on the transcript. The ratio of these probabilities under the biased model to the uniform (null) model is represented by  and , respectively.

The parameters that need to be estimated in the likelihood function are the abundances *ρ *and the bias parameters described above. We estimate the parameters using coordinate ascent. The model is linear in the *ρ *parameters for fixed bias parameters, and we maximize them as in [6]. For fixed *ρ*, the bias parameters can be maximized as described in the Supplementary methods in Additional file 3. Therefore, we employ an iterative coordinate ascent procedure that, in effect, jointly maximizes all parameters. We found, however, that the gains in likelihood after the first iteration do not justify the time requirements, and we therefore limit all experiments to a single iteration. An initial *ρ*_0 _estimation with uniform bias weights seeds the maximization of the bias parameters. *ρ *is then maximized using these bias parameters, and is used as the final abundance estimate.

### NanoString experiment

Cell culture/RNA prep and NanoString: Mouse embryonic stem cells (V6.5) were co-cultured with irradiated mouse embryonic broblasts as described in [26]. mESCs were passaged once on gelatin alone before RNA extraction. Total RNA was extracted from mESCs using the protocol specified in the RNeasy kit (Qiagen). 100 ng of total RNA was hybridized for 17 hours with lincRNA codeset in technical triplicate. The hybridized material was loaded into the nCounter prep station followed by quantification on the nCounter Digital Analyzer as outlined by NanoString Technologies in their Total RNA Gene Expression nCounter protocol.

### RNA-Seq data

All accession numbers refer to data available from the short read archive (SRA) [27]. The SOLiD data was downloaded from [28]. The NSR data was provided by the authors of [21].

### Mapping and annotation

To allow for consistent comparison across datasets, all read mapping was carried out using TopHat 1.1.0 [29] with supplied annotations and the --no-novel-juncs option set, except for the SOLiD datasets, which were only available in a pre-aligned form with mapping by BioScope 1.2.1. All expression estimation and bias correction were done using Cufflinks 0.9.3 with the same annotation and reference sequence as TopHat. In the case of strand-specific libraries, the correct --library-type option was used as per the Cufflinks manual. For the mouse dataset in the NanoString experiment, the RefSeq refGene annotation for assembly NCBI37/mm9 was used, and was downloaded from the UCSC Genome Browser. For all human datasets, the RefSeq refGene annotation for assembly NCBI36/hg18 [30] was used, and was downloaded from the UCSC Genome Browser. The only filtering was to remove non-chromosomal and 'random' contigs. After quanti cation with Cufflinks, the subset of transcripts with 1-to-1 mappings to the TaqMan qRT-PCR probes were selected (as listed in the supplement to [16]) to be used in the correlation tests. All yeast datasets used the *Ensembl Saccharomyces cerevisiae *annotation, release 59, which was downloaded from the Ensembl website [31]. Mitochondrial, non-coding, and ribosomal RNA sites were masked in the annotation. All remaining transcripts were used in our correlation tests.

### Comparison with previous methods

#### Genominator

We downloaded the Genominator package version 1.4.0 using Bioconductor. We then followed the instructors provided with the Genominator for 'Working with the ShortRead Package'. We used the same annotations as in our Cufflinks experiment to define the ranges and transcript lengths for the RPKM calculations. We also used the same read mapping as was used for Cufflinks. Due to memory limitations of the software, we were forced to learn the weights separately for each chromosome.

#### mseq

We downloaded the mseq package version 1.1 from the Comprehensive R Archive Network (CRAN). Due to the specific mseq input format requirements, we remapped the reads using Bowtie version 0.12.5 [32] with the --best option and default parameters otherwise. The mapping was then converted to the mseq input format using a custom Python script we wrote and that is provided in Additional file 4. We followed the instructions of [10] and trained the MART model with suggested parameters on the top 100 expressed transcripts, which we determined by computing the RPKM for every transcript. The UTR regions and an additional 100 bases on the ends of transcripts were excluded from the training. The surrounding sequence window was set to be 8 bases before and 12 bases after the first nucleotide in the read, which matches the window of our variable length Markov model and is where we observed bias for the dataset. The resulting sequence preferences were used to find the corrected RPKMs.

### Statistical analysis

All correlation tests used least squares linear regression, as implemented in the R programming language. We found the *P*-value in Section 2.2 by sampling (with replacement) from the empirical distribution of fold changes 50,000 times for each transcript in order to generate 50,000 randomly adjusted sets of expression values. Of these, only 35 showed correlations better than the values that were corrected by our method (*R*^2 ^= 0.81).

## Abbreviations

bp: base pair; CRAN: Comprehensive R archive network; FPKM: Fragments per kilobase per million reads sequenced; HBR: Human brain reference; MAQC: Microarray quality control; NNSR: Not not so random (hexamer priming); NSR: Not so random (hexamer priming); qRT-PCR: Quantitative reverse transcription polymerase chain reaction; RH: Random hexamer (priming); RPKM: Reads per kilobase per million reads sequenced; SRA: Short read archive; UHR: Universal human reference; WT: whole transcriptome.

## Authors' contributions

AR, CT and LP developed the bias correction approach. AR implemented the improvements to the Cufflinks software. JLR provided reagents and guidance. JD performed the NanoString experiment. AR performed the analysis. AR and LP wrote the paper. All authors read and approved the final manuscript.

## Supplementary Material

Additional file 1 **Supplementary figures**. Additional figures referred to in the text. Click here for file

Additional file 2 **Raw expression data**. Raw expression estimates used in comparisons, including the NanoString expression measurements. Click here for file

Additional file 3 **Supplementary methods**. More detail on the likelihood model. Click here for file

Additional file 4 **Script for ****mseq**** format conversion**. Python script that converts standard SAM and FASTA input into the mseq input format. Click here for file
