# Supplementary material for: Improving RNA-Seq expression estimates by correcting for fragment bias
Source: Genome Biol. 2011 Mar 16;12(3):R22. doi: 10.1186/gb-2011-12-3-r22 (PMC3129672; doi:10.1186/gb-2011-12-3-r22)
Supplement: Additional file 1 — Supplementary figures. Additional figures referred to in the text. [file gb-2011-12-3-r22-S1.PDF]

Supplementary Figure S1

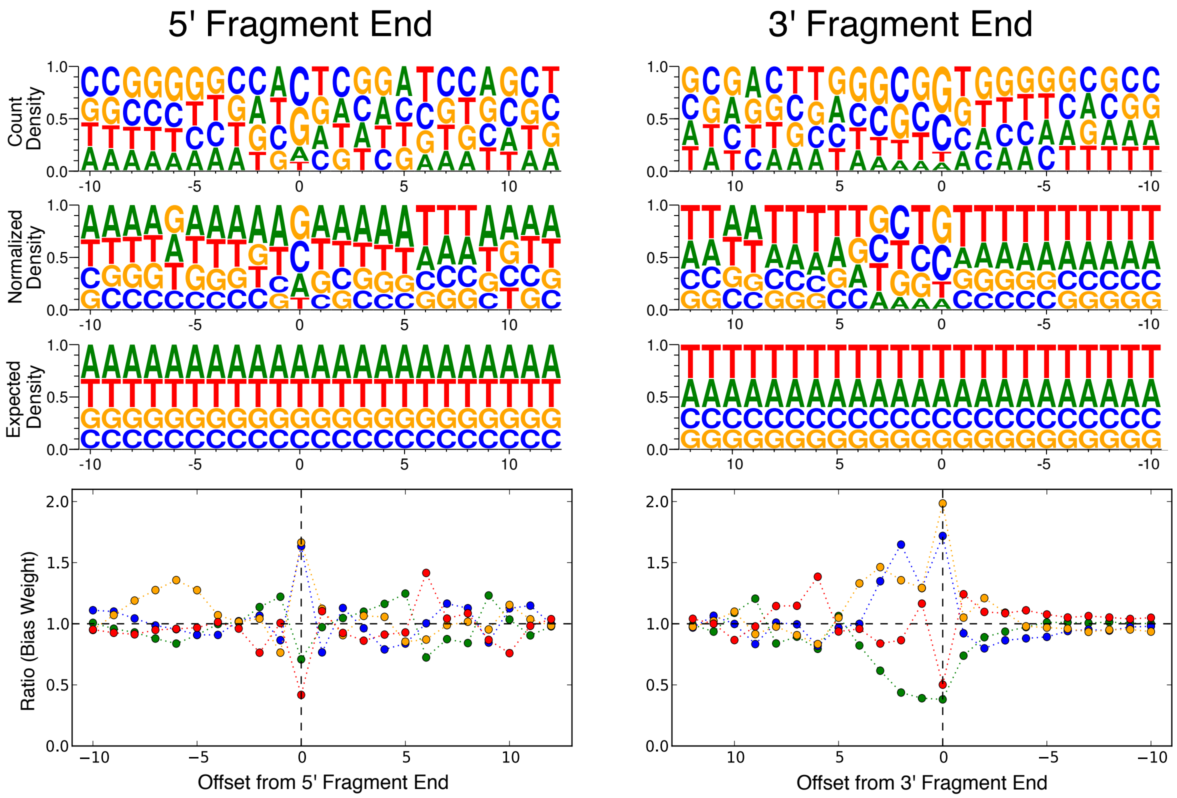

This plot shows nucleotide frequencies surrounding the fragment ends for the control experiment in Levin, et al 2010. Note that the 3' sequences are complemented in order to represent that nucleotides that are being primed in second-strand synthesis. See Figure 2 in the main text for more details.

Supplementary Figure S2

The panels below show the inferred bias for each experiment mentioned in the main text. The first can be used as a legend to help interpret the meaning of each plot. Note that the interpreation of the plots in the second row of each figure is identical to Figure 2 (D) of the main text.

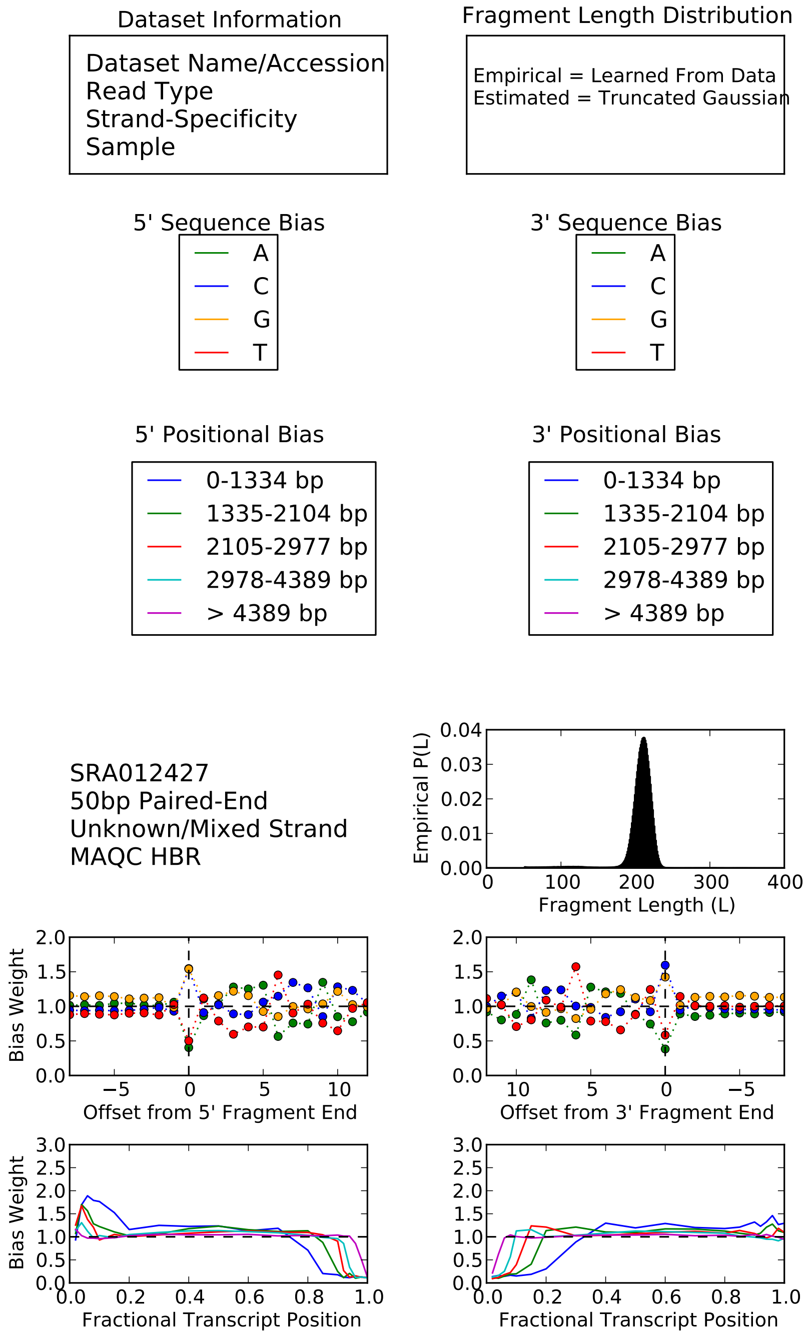

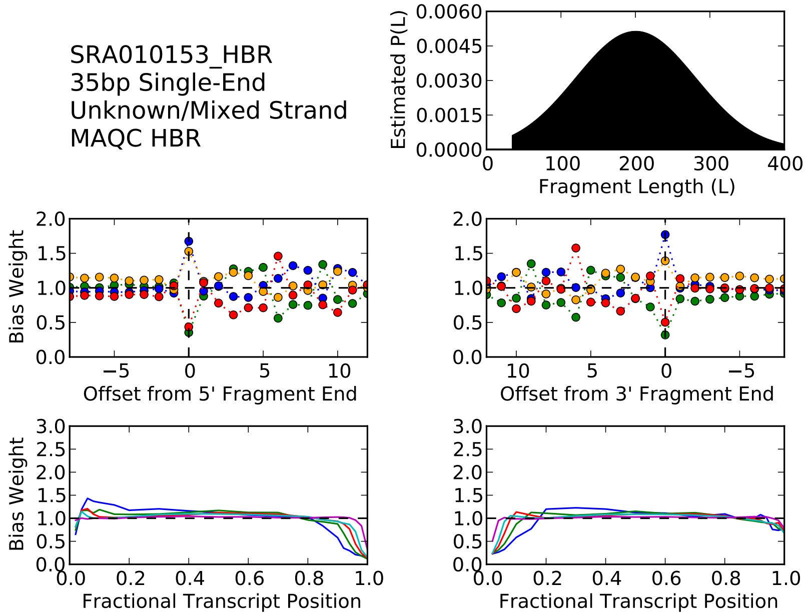
SRA010153_HBR
35bp Single-End
Unknown/Mixed Strand
MAQC HBR
Estimated P(L)
Fragment Length (L)
Bias Weight
Offset from 5' Fragment End
Offset from 3' Fragment End
Fractional Transcript Position

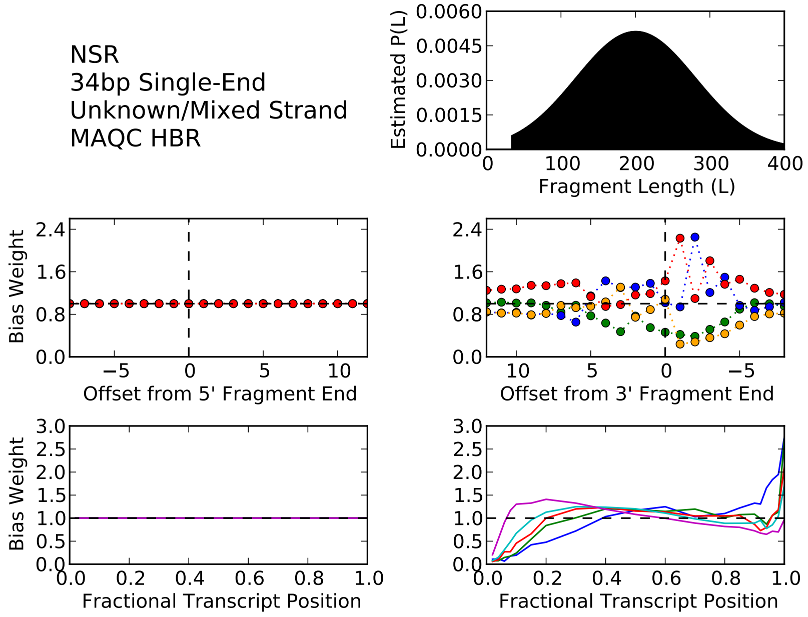
NSR
34bp Single-End
Unknown/Mixed Strand
MAQC HBR
Estimated P(L)
Fragment Length (L)
Bias Weight
Offset from 5' Fragment End
Offset from 3' Fragment End
Fractional Transcript Position

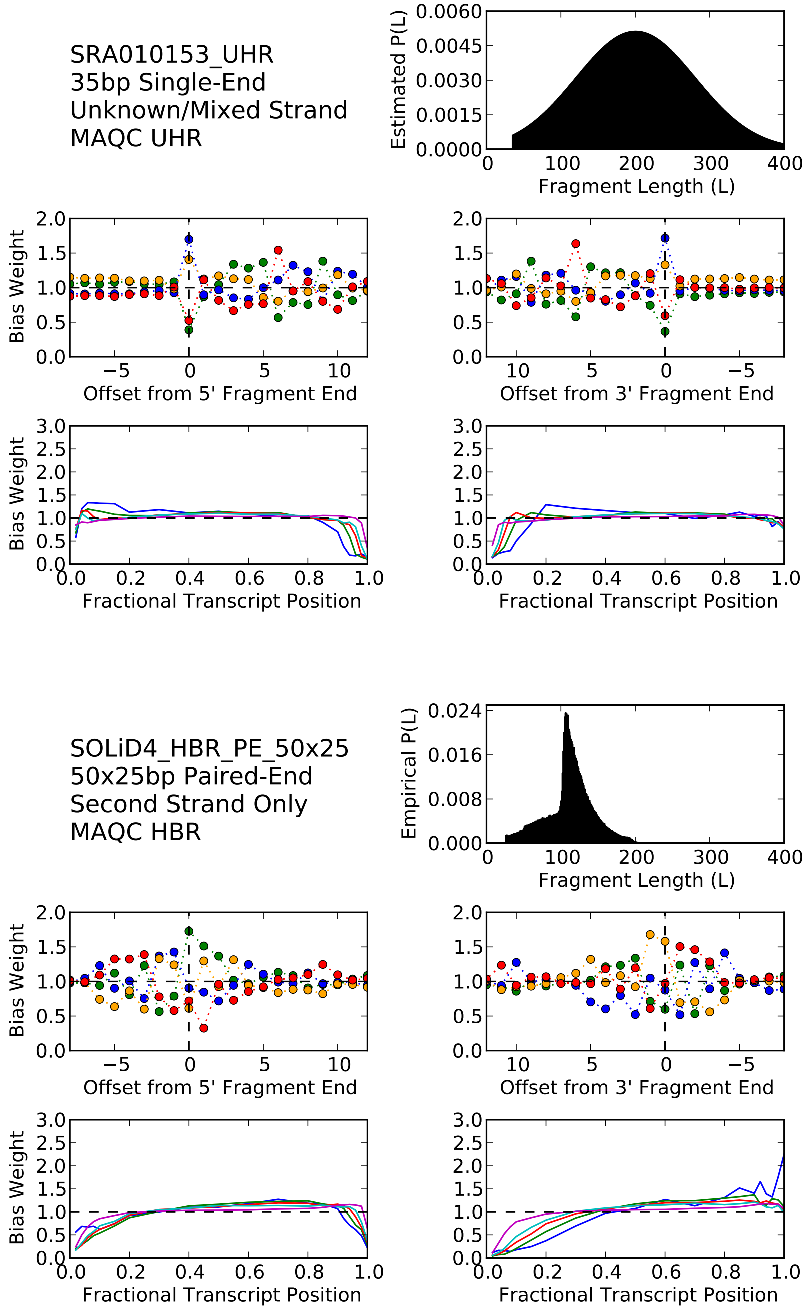
SRA010153_UHR
35bp Single-End
Unknown/Mixed Strand
MAQC UHR
Estimated P(L)
Fragment Length (L)
Bias Weight
Offset from 5' Fragment End
Offset from 3' Fragment End
Fractional Transcript Position
SOLiD4_HBR_PE_50x25
50x25bp Paired-End
Second Strand Only
MAQC HBR
Empirical P(L)

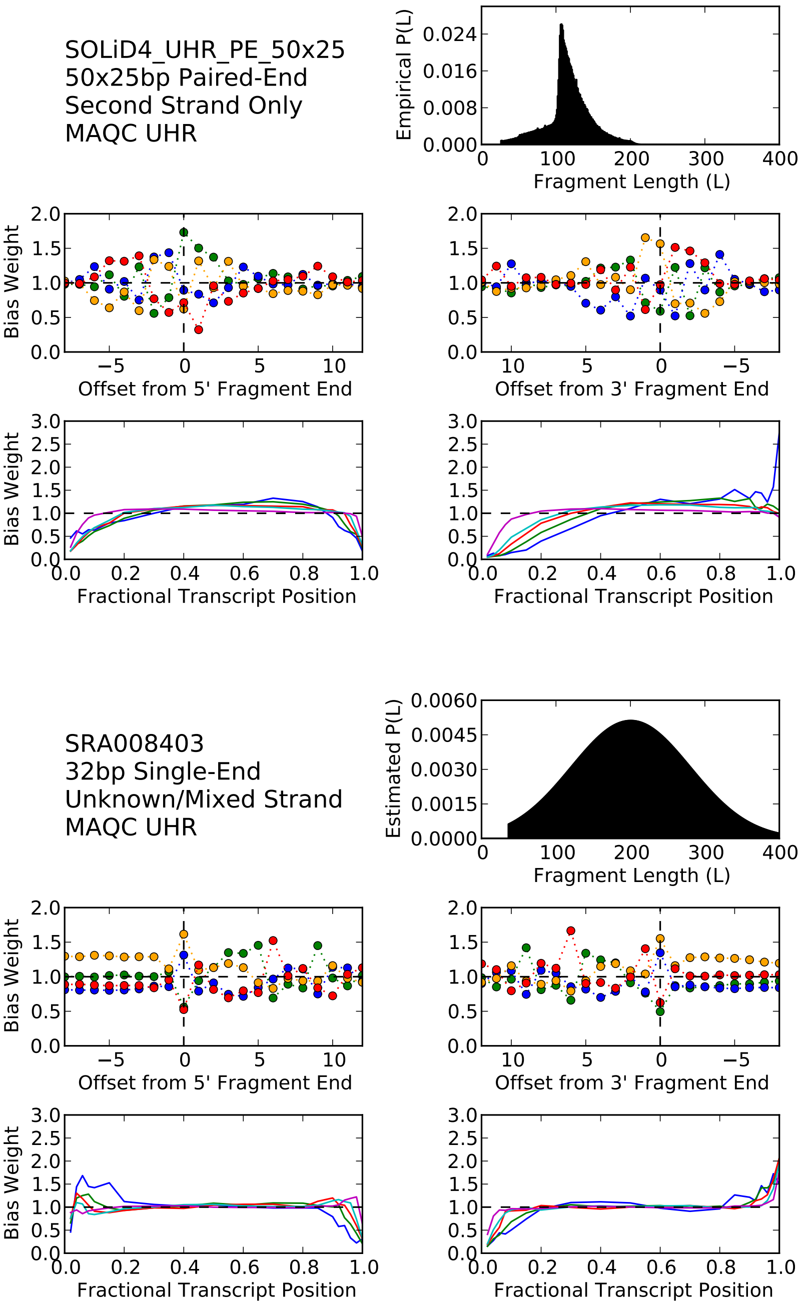
SOLiD4_UHR_PE_50x25
50x25bp Paired-End
Second Strand Only
MAQC UHR
Empirical P(L)
Fragment Length (L)
Bias Weight
Offset from 5' Fragment End
Offset from 3' Fragment End
Fractional Transcript Position
SRA008403
32bp Single-End
Unknown/Mixed Strand
MAQC UHR
Estimated P(L)
Fragment Length (L)
Bias Weight
Offset from 5' Fragment End
Offset from 3' Fragment End
Fractional Transcript Position

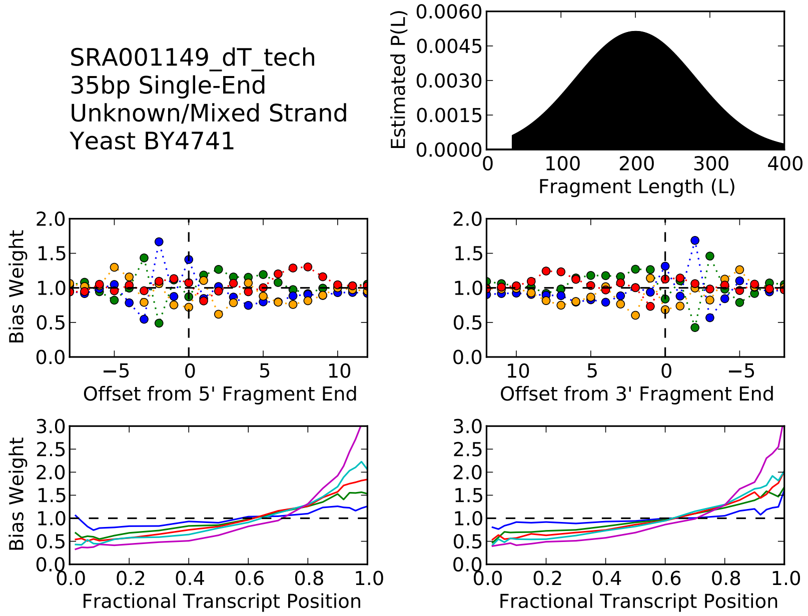
SRA001149_dT_tech
35bp Single-End
Unknown/Mixed Strand
Yeast BY4741
Estimated P(L)
Fragment Length (L)
Bias Weight
Offset from 5' Fragment End
Offset from 3' Fragment End
Fractional Transcript Position

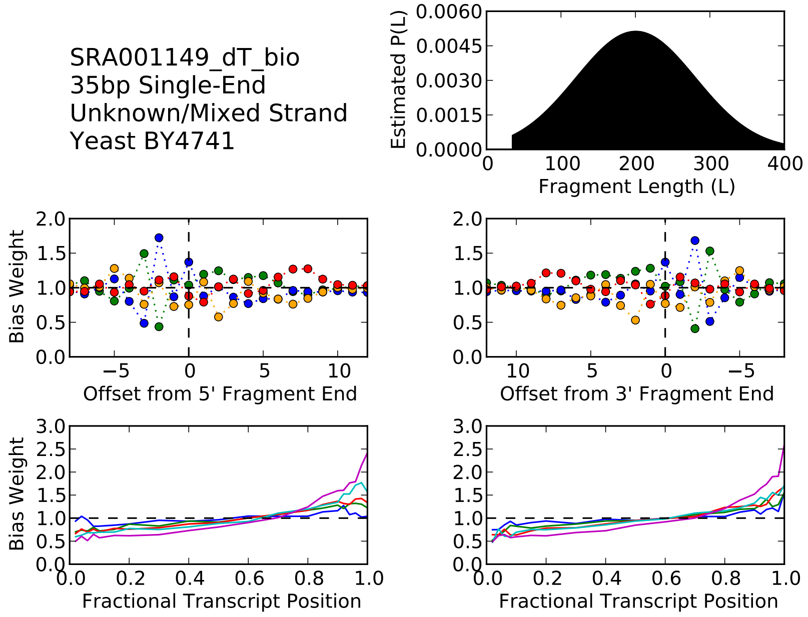
SRA001149_dT_bio
35bp Single-End
Unknown/Mixed Strand
Yeast BY4741
Estimated P(L)
Fragment Length (L)
Bias Weight
Offset from 5' Fragment End
Offset from 3' Fragment End
Fractional Transcript Position

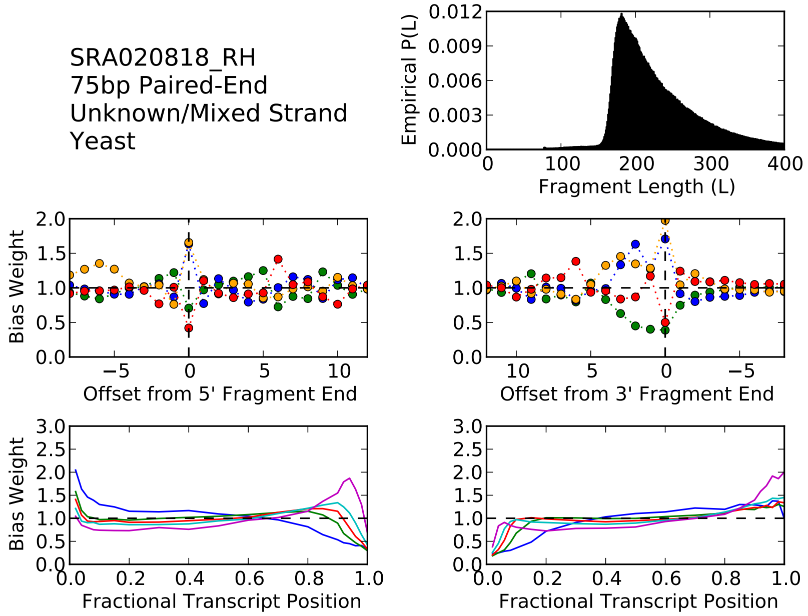
SRA020818_RH
75bp Paired-End
Unknown/Mixed Strand
Yeast
Empirical P(L)
Fragment Length (L)
Bias Weight
Offset from 5' Fragment End
Offset from 3' Fragment End
Bias Weight
Fractional Transcript Position
Fractional Transcript Position

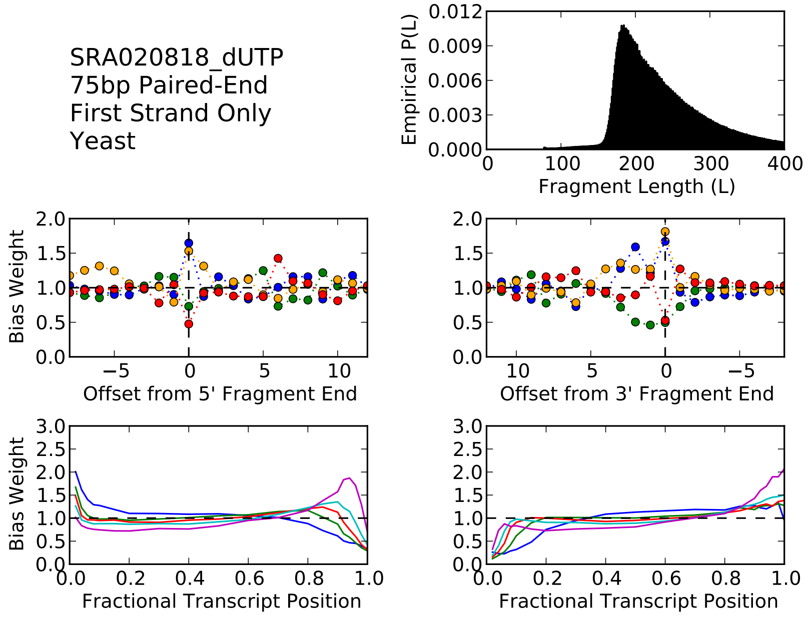
SRA020818_dUTP
75bp Paired-End
First Strand Only
Yeast
Empirical P(L)
Fragment Length (L)
Bias Weight
Offset from 5' Fragment End
Offset from 3' Fragment End
Bias Weight
Fractional Transcript Position
Fractional Transcript Position

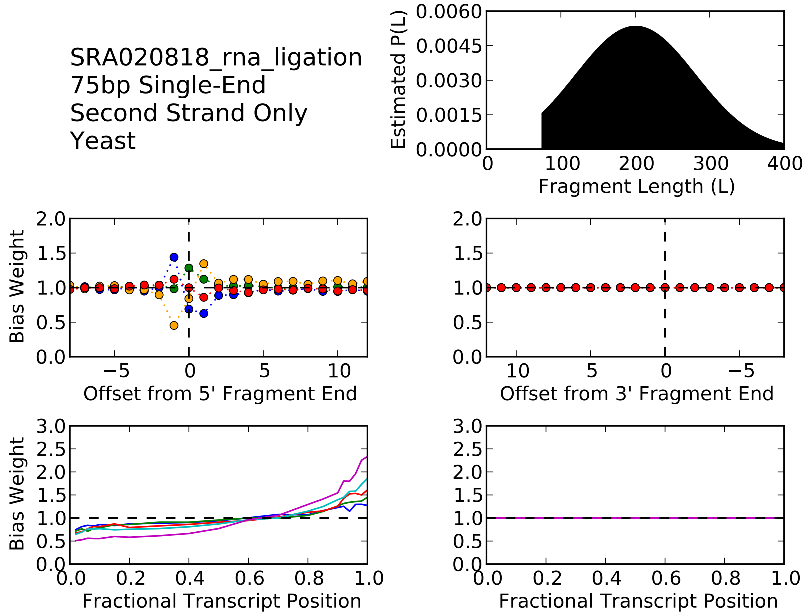
SRA020818_rna_ligation
75bp Single-End
Second Strand Only
Yeast
Estimated P(L)
0.0060
0.0045
0.0030
0.0015
0.0000
0
100
200
300
400
Fragment Length (L)
Bias Weight
2.0
1.5
1.0
0.5
0.0
−5
0
5
10
Offset from 5' Fragment End
10
5
0
−5
Offset from 3' Fragment End
3.0
2.5
2.0
1.5
1.0
0.5
0.0
0.2
0.4
0.6
0.8
1.0
Fractional Transcript Position
Fractional Transcript Position

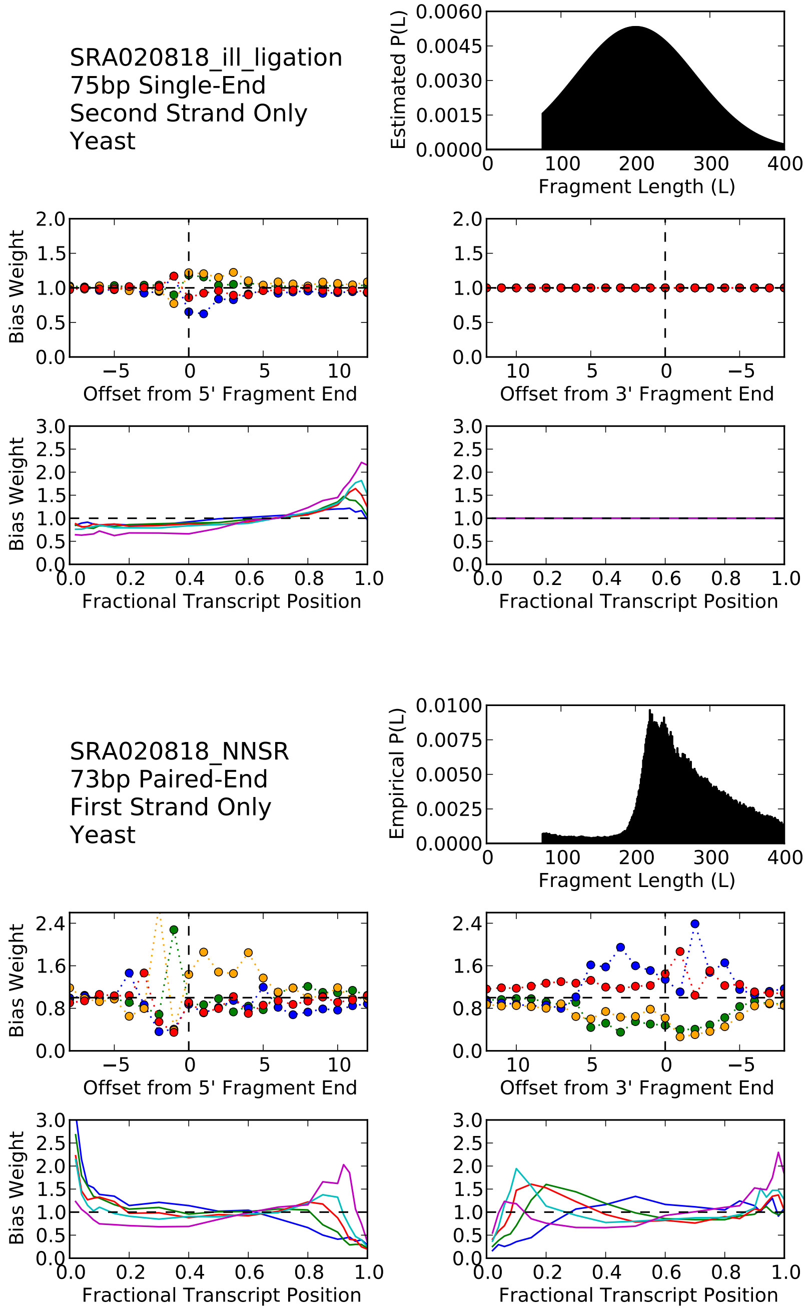
SRA020818_ill_ligation
75bp Single-End
Second Strand Only
Yeast
Estimated P(L)
Fragment Length (L)
Bias Weight
Offset from 5' Fragment End
Offset from 3' Fragment End
Fractional Transcript Position
SRA020818_NNSR
73bp Paired-End
First Strand Only
Yeast
Empirical P(L)
Fragment Length (L)
Bias Weight
Offset from 5' Fragment End
Offset from 3' Fragment End
Fractional Transcript Position

Supplementary Figure S3

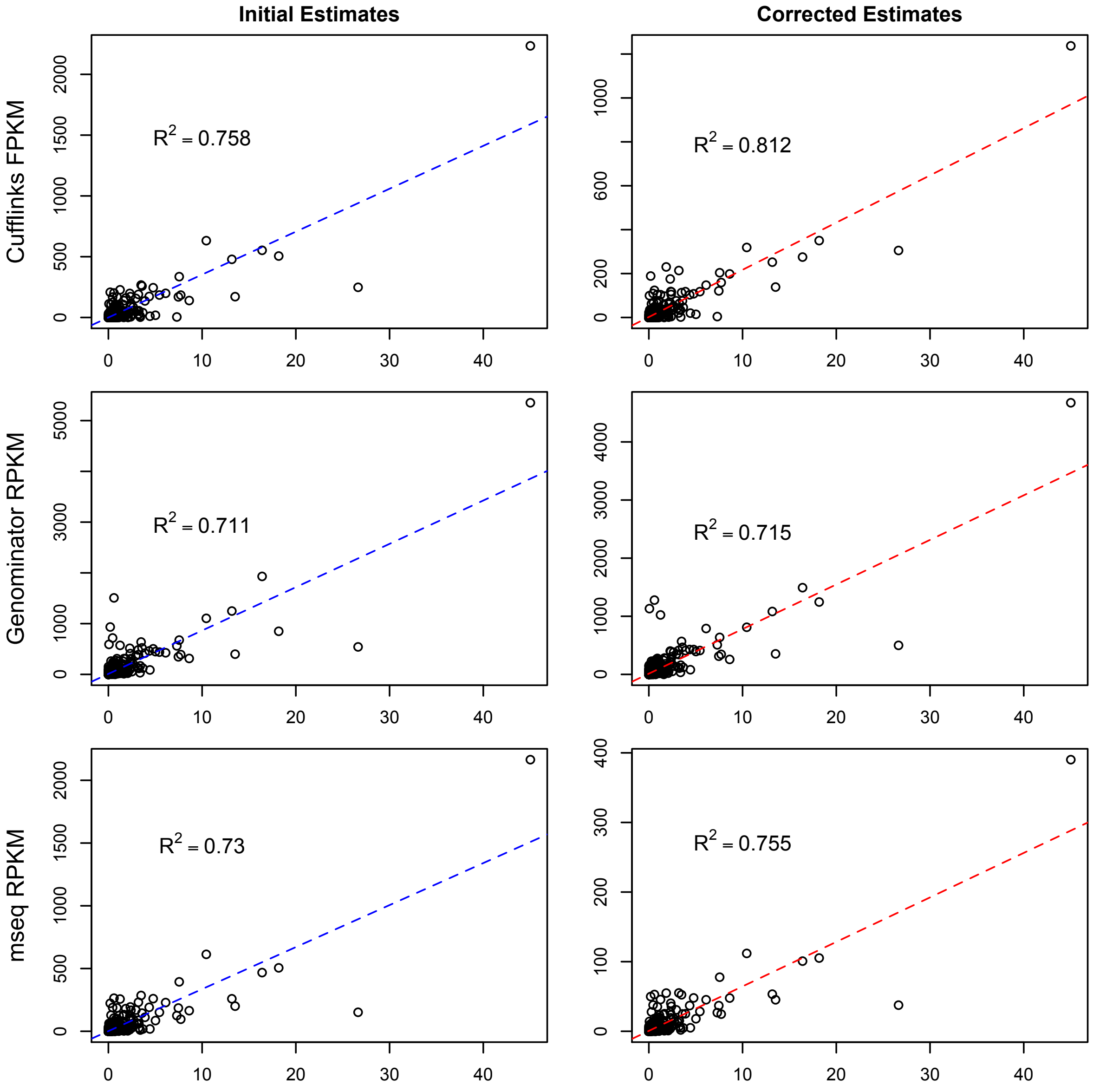

Plots showing the correlation between the TaqMan qPCR data and RNA-Seq expression estimates before (left) and after (right) the three correction methods compared in the text.

Supplementary Figure S4

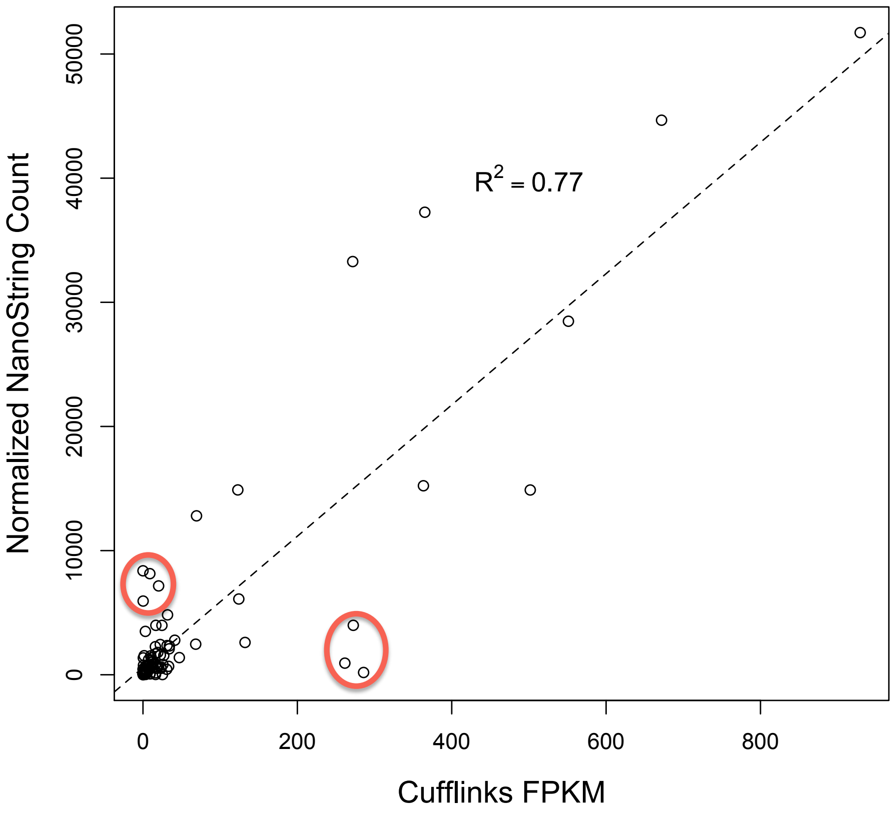

We compared our expression estimates to NanoString on a set of 95 genes, where for each gene we performed a NanoString experiment (see Methods). Although the overall correlation was good ($R^2 = 0.77$), we could not explain a number of outliers (circled), and we also did not find an improvement in correlation when correcting for bias (in contrast to the case with qRT-PCR that we elaborate on in the main text and all other validations we attempted). Furthermore, we noticed high variance between replicates (see Data). We report these data because of its value in assessing expression accuracy in conjunction with previously generated data reported in Trapnell et al. 2010.
