# Supplementary material for: Improving RNA-Seq expression estimates by correcting for fragment bias
Source: Genome Biol. 2011 Mar 16;12(3):R22. doi: 10.1186/gb-2011-12-3-r22 (PMC3129672; doi:10.1186/gb-2011-12-3-r22)
Supplement: Additional file 3 — Supplementary methods. More detail on the likelihood model. [file gb-2011-12-3-r22-S3.PDF]

SUPPLEMENTARY METHODS FOR THE PAPER IMPROVING RNA-SEQ EXPRESSION ESTIMATES BY CORRECTING FOR FRAGMENT BIAS

ADAM ROBERTS, COLE TRAPNELL, JULIE DONAGHEY, JOHN L. RINN, AND LIOR PACHTER

1. Likelihood Model for RNA-Seq

1.1. **Preliminaries.** We begin by describing our model for paired-end fragments, in which case the data consists of a set of paired-end fragments F, as well as a genomic reference sequence S, transcript annotation G, and an initial abundance estimation ρ_t^0 for each transcript. We annotate the transcriptome and compute the initial abundance estimates as in [6] without bias estimation. The fragments $f \in F$ are defined as tuples containing the $5'$ and $3'$ ends of fragments in genomic coordinates. We define a pair of functions $e_{5'}(t,f)$ and $e_{3'}(t,f)$, which return the ends of each of the fragments $f \in F$ in *transcript* coordinates (here t can be any transcript compatible with f). Let $l(t)$ be the length of transcript t and $I_t(f)$ be the inferred length of fragment f in reference to transcript t, which we will define as

$$I_t(f) \equiv |e_{5'}(t,f) - e_{3'}(t,f)| + 1.$$

For a given probability distribution function (pdf) and cumulative distribution function (cdf) of the fragment length distribution, we denote by $D(t,f)$the probability of fragment f being its inferred length for t based on its starting position within t, assuming that each starting position has an equal probability of a fragment starting at it (excluding positions less than a minimum read length from the end of the transcript). Therefore, $D(t,f)$ is obtained by renormalizing the fragment length pdf by the remaining possible fragment lengths from the start site of f, which can be written as

$$D(t,f) = \frac{\text{pdf}(I_t(f))}{\text{cdf}(l(t) - e_{5'}(t,f) + 1)}.$$

1.2. **Likelihood Function.** We can now modify the likelihood of [6] to refer to fragments instead of reads, as follows.

$$\begin{aligned}
L(\rho^1|F,G) &= \prod_{f\in F}\mathbb{P}(frag.\ aln. = f) \\
&= \prod_{f\in F}\sum_{g\in G}\mathbb{P}(frag.\ aln. = f|locus = g)\mathbb{P}(locus = g) \\
&= \prod_{f\in F}\frac{\sigma_{g_f}\tilde{l}(g_f)}{\sum_{g\in G}\sigma_g\tilde{l}(g)}\mathbb{P}(frag.\ aln. = f|locus = g_f) \\
&= \prod_{f\in F}\beta_{g_f}\sum_{t\in g_f}\mathbb{P}(frag.\ aln. = f|locus = g_f, trans. = t)\mathbb{P}(trans. = t|locus = g_f) \\
&= \prod_{f\in F}\beta_{g_f}\sum_{t\in g_f}\frac{\tau_t\tilde{l}(t)}{\sum_{u\in g_f}\tau_u\tilde{l}(u)}\mathbb{P}(frag.\ aln. = f|locus = g_f, trans. = t) \\
&= \left(\prod_{f\in F}\beta_{g_f}\right)\left(\prod_{f\in F}\sum_{t\in g}\gamma_t\cdot\mathbb{P}(frag.\ aln. = f|locus = g_f, trans. = t)\right).
\end{aligned}$$

Removing the assumption of uniform read coverage within a transcript requires a change in the definition of $\mathbb{P}(frag.\ aln. = f|locus = g_f, trans. = t)$, which was formerly assumed to be $D(t,f)\frac{1}{l(t)-I_t(f)-1}$ (with the new notation). We now wish to associate a bias weight with each possible fragment deriving from a given transcript. We will define such a bias weight as $b(t,i,j)$, where i is the index of the $5'$ end and j is the index of the $3'$ end in transcript t. For now, we will ignore how we calculate this actual weight and continue our derivation of the likelihood function by defining the total bias of a transcript for a given fragment length L as

$$B(t,L) = \sum_{i=1}^{l(t)-L+1} b(t,i,i+L-1).$$

Therefore,

$$\mathbb{P}(frag.\ aln. = f|locus = g_f, trans. = t) = D(t,f)\frac{b(t,e_{5'}(t,f),e_{3'}(t,f))}{B(t,I_t(f))}.$$

The likelihood equation forms the basis for estimating relative transcript abundances within loci (the γ parameters), but there is also an adjustment needed to incorporate bias estimation into overall transcript abundance estimates. The overall expression for a transcript ρ_t is reported in FPKM units (see [6]) and is given by

$$FPKM_t = \frac{10^9 X_g\hat{\gamma}_t}{\tilde{l}(t)M}$$

where $\hat{\gamma}_t$ is the maximum likelihood estimate for the relative abundance of transcript t within locus g, M is the total number of fragments sequenced, and

$$\tilde{l}(t) = \sum_{L=1}^{l(t)} D(t,(1,L+1))B(t,L).$$

is the *effective length* of transcript t. Intuitively, the effective length can be thought of as a measure of the number of sites at which a fragment can start in a given transcript. In the unbiased case, and assuming fragments have the same length, a transcript will have an effective length equal to the transcript length minus the fragment length, since each interior site would be equally likely to start a fragment. In the biased case, this measure may be smaller or larger, depending on the bias associated with sequences present in the transcript (sequences with higher bias weight leading to a transcript that is effectively longer). In this sense, we can use the fold change in effective length after bias correction as a measure of the total bias in a transcript.

2. Bias Weights

Assuming the bias at the ends of a fragment are independent, we will define $b_{5'}(t,i)$ and $b_{3'}(t,j)$ to be separate bias weights and let $b(t,i,j) = b_{5'}(t,i) \cdot b_{3'}(t,j)$. We further separate the bias weights into the two types: sequence-specific $(b^s_{5'}, b^s_{3'})$ and positional $(b^p_{5'}, b^p_{3'})$.

2.1. Sequence-Specific Weights.

Let $seq_{t,i}$ denote the sequence in a window surrounding position i in transcript t. The weight for the $5'$ (w.l.o.g.) sequence-specific bias at a position i in transcript t is

$$b^s_{5'}(t,i) = \frac{\mathbb{P}(seq_{t,i}|F,G,\rho_0)}{\mathbb{P}(seq_{t,i}|G,\rho_0)}$$

In words, this is the ratio of the probability of the sequence given the fragment alignments to the probability of the sequence given the annotation and abundances with the assumption of uniform coverage of transcripts (the null model). We now must derive a manner in which to calculate these probabilities.

We start by defining the window to be from 8bp upstream of the $5'$ end to 11bp downstream. For the $3'$ end, we take the reverse complement of the sequence and define the window similarly. This window was chosen based on plots such as Figure 1, which show that the bias does not usually extend beyond this region, aside from codon frame selection effects. To limit the number of parameters we must learn while allowing a large window size, we use a sparse DAG representation with maximum edge length 3. We use the same DAG with different parameters for both the null and biased model, as well as the $5'$ and $3'$ ends. Thus, the probabilities factorize as the product of conditional probability parameters of our DAG, which we will label as $\psi^{5';0}_{n,\pi_n}$ for the $5'$ null (0) model (1 in the biased model), where n is the node (or position in the window) and π_n are the

parent nodes of node n in our DAG. Therefore,

$$b^s_{5'}(t,i) = \frac{\prod_{n=1}^{21} \psi^{5',1}_{n,\pi_n}}{\prod_{n=1}^{21} \psi^{5',0}_{n,\pi_n}} = \prod_{n=1}^{21} \frac{\psi^{5',1}_{n,\pi_n}}{\psi^{5',0}_{n,\pi_n}}$$

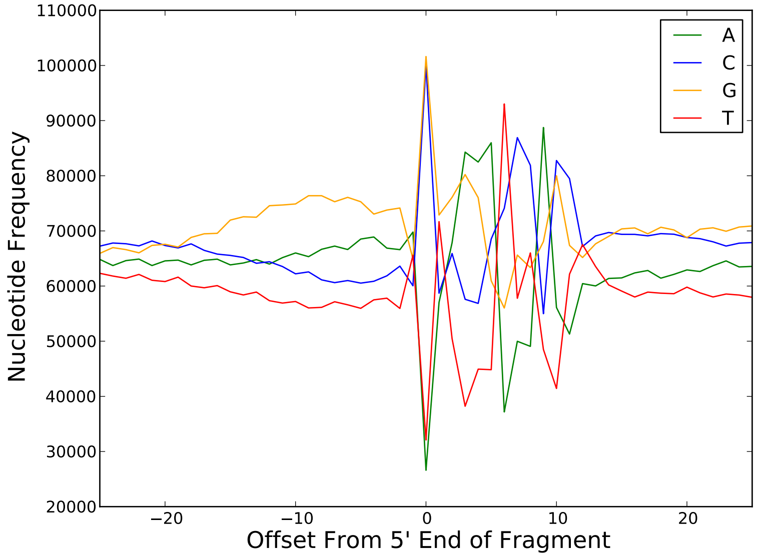

FIGURE 1. Nucleotide frequencies in 51bp window surrounding the $5'$ end of fragments from the Illumina HBR dataset (SRA012427) described in the main text. Only reads mapping to single isoform genes are included and counts are corrected for abundance by weighting the contribution of each fragment by the initial (not bias corrected) FPKM of the gene it is mapped to. Note that much of the bias lies within the 21bp window we have chosen (8bp upstream to 11bp downstream of the fragment end at offset 0). Also note that a reading frame is preferentially selected leading to a repeated 3bp motif even outside of the 21bp window. This phenomena is prevalent in the datasets we have examined.

There are now two sets of parameters that must be estimated, the π parameters that define our DAG and the ψ parameters that specify the conditional probabilities separately for the $5'$ and $3'$ ends under the uniform (null) and biased models. The maximization of the ψ parameters for a fixed DAG are explained below. For the π parameters, we implemented a hill climbing procedure to maximize the likelihood under the above assumptions. We found that the resulting DAG did not vary much among datasets, and while the qPCR correlation validations were greatly improved by including the correlation between positions in our model, little was lost by using a sub-optimal DAG. Therefore, we chose a VLMM (Figure 2) that kept the number of parameters low while retaining the important correlations, allowing us to save time on per-experiment model selection with very little loss in explanatory power.

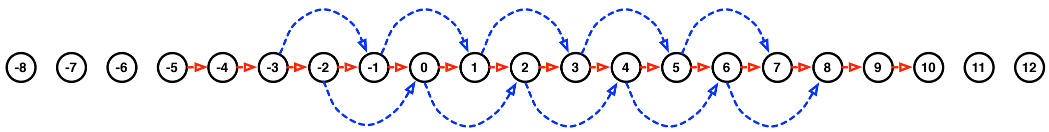

FIGURE 2. The VLMM used to model sequence-specific bias. The node indexes represent the offset from the first base in the read sequence (0). Red arrows show first order dependencies, and blue second-order. This VLMM requires 744 parameters in order to be fully specified.

2.2. **Positional Weights.** To specify the positional bias weights, we again wish to calculate a ratio of probabilities, which for the $5'$ (w.l.o.g.) end is

$$b^p_{5'}(t,i) = \frac{\mathbb{P}(f_{5'} = i | l(t), F, G, \rho_0)}{\mathbb{P}(f_{5'} = i | l(t), G, \rho_0)}$$

To reduce the number of parameters we need to estimate these probabilities, we use relative positions within a transcript and bin these into 20 bins for 5 transcript lengths (see legend in Figure S2 of Additional file 2). We label these parameters for the $5'$ end under the null model (w.l.o.g.) as $\omega^{5',0}_{L,r}$ where L is the transcript length and r is the bin for the relative position. Therefore,

$$b^p_{5'}(t,i) = \frac{\omega^{5,1}_{l(t),i/l(t)}}{\omega^{5,0}_{l(t),i/l(t)}}$$

3. Bias Parameter Estimation

In the main text we describe how bias parameters are estimated using fixed ρ. To make this maximization tractable, we do not not use the likelihood function above but instead make some simplifying approximations to speed up computations. Since the sequence-specific bias arises during the priming for reverse transcription, we assume that the positions in the RNA fragments are selected for priming out of the pooled set of all other fragments, not just those within the same transcript. We also assume that the priming of the ends are independent events.

These assumptions allow us to maximize the ψ and ω by taking proportions that are the maximum likelihood estimates for log-linear models instead of having to resort to numerical procedures.

Furthermore, since we must take into account relative abundances when learning the parameters, we limit our learning to single isoform genes since their abundance estimates (ρ values) will be more accurate after the first estimation step.

4. Determining $5'$ and $3'$ Fragment Ends

It is important to be able to determine which end of the fragment a given read is derived from ($5'$ or $3'$) since the bias is clearly different for a protocol depending on whether the end was initially primed in first-strand synthesis or second-strand synthesis. Whenever available, a few key pieces of information allow us to solve this problem with

certainty so that we can correctly estimate our parameters. For a paired-end fragment we can label the ends if we know whether the fragment itself was generated during first- or second-strand synthesis. This is because standard DNA sequencers (such as Illumina and SOLiD) sequence pairs in a "forward-reverse" orientation, meaning that the the first read in the pair aligns to the actual fragment being sequenced and derives from its $5'$ end, while the second is the reverse complement of the 3' end. If it is known that all of the sequenced fragments were generated in first-strand synthesis (as in the dUTP and NSR protocols), then the first read in the pair will always be the $3'$ end (in transcript orientation) of the fragment. If it is known that all sequenced fragments were generated in second-strand synthesis (as in the ligation and SOLiD protocols), then the first read in the pair will always be the $5'$ end. Moreover, these rules trivially apply to single-end reads as that situation is equivalent to only knowing the "first sequenced" end of a paired-end experiment. Clearly, we could also determine the ends if a mixture of first- and second-strand synthesis fragments were sequenced as long as we are provided with some information that reveals which stage each fragment was generated during. One such situation (in the absence of a strand-specific protocol) is when the orientation of the transcript that the fragment is derived from is known. This information might be provided in a known annotation, or it can be inferred from another read spanning a splice junction on the same transcript. If the first read in a fragment aligns to the sense DNA strand of its transcript, then the first read must be the 3' end of the fragment, or, equivalently, the read was generated during first-strand synthesis (see Figure 1 in main text). The reverse is true if the first read aligns to the antisense DNA strand. The key is that the sense and antisense strand for a given transcript must be known a priori or be inferred from splice reads. As with strand-specific protocols, these rules also extend to the case of single-end reads.
